# Squamous differentiation requires G2/mitosis slippage to avoid apoptosis

**DOI:** 10.1038/s41418-020-0515-2

**Published:** 2020-02-20

**Authors:** Natalia Sanz-Gómez, Isabel de Pedro, Beatriz Ortigosa, David Santamaría, Marcos Malumbres, Guillermo de Cárcer, Alberto Gandarillas

**Affiliations:** 1 Cell Cycle, Stem Cell Fate and Cancer Laboratory, Institute for Research Marqués de Valdecilla (IDIVAL), 39011 Santander, Spain; 2 Cell Cycle & Cancer Biomarkers Group, Instituto de Investigaciones Biomédicas “Alberto Sols” (IIBm) CSIC-UAM, 28029 Madrid, Spain; 3 CNIO, Experimental Oncology Group, Spanish National Cancer Research Centre (CNIO), 28029 Madrid, Spain; 4 CNIO, Cell Division and Cancer Group, Spanish National Cancer Research Centre (CNIO), 28029 Madrid, Spain; 5 INSERM, Languedoc-Roussillon, 34394 Montpellier, France; 6 Present Address: INSERM U1218, ACTION Laboratory, IECB, University of Bordeaux, Pessac, France

**Keywords:** Physiology, Cancer

## Abstract

The cellular mechanisms controlling cell fate in self-renewal tissues remain unclear. Cell cycle failure often leads to an apoptosis anti-oncogenic response. We have inactivated Cdk1 or Polo-like-1 kinases, essential targets of the mitotic checkpoints, in the epithelia of skin and oral mucosa. Here, we show that inactivation of the mitotic kinases leading to polyploidy in vivo, produces a fully differentiated epithelium. Cells within the basal layer aberrantly differentiate and contain large or various nuclei. Freshly isolated KO cells were also differentiated and polyploid. However, sustained metaphase arrest downstream of the spindle anaphase checkpoint (SAC) due to abrogation of CDC20 (essential cofactor of anaphase-promoting complex), impaired squamous differentiation and resulted in apoptosis. Therefore, upon prolonged arrest keratinocytes need to slip beyond G2 or mitosis in order to initiate differentiation. The results altogether demonstrate that mitotic checkpoints drive squamous cell fate towards differentiation or apoptosis in response to genetic damage.

## Introduction

The mechanisms coordinating proliferation, differentiation and apoptosis in self-renewal tissues are still intriguing. DNA damage is known to induce apoptosis or senescence as a cellular response to the oncogenic potential of genetic alterations [1]. DNA damage triggers G2 and mitosis checkpoints what subsequently results in mitotic catastrophe [2, 3]. The control of cell fate upon DNA damage is important to homoeostasis and morphogenesis, even more in self-renewal tissues that are highly exposed to genotoxic agents.

Squamous-stratified epithelia are present in a variety of mammalian locations: skin, oral cavity, larynx, pharynx, oesophagus, cervix and vagina. They are developing tissues under continuous self-renewal, and they are continuously exposed to mutational injury. Given the continuous genetic impact they receive (UV irradiation, alcohol, tobacco and HPV), they must have robust mechanisms to maintain homoeostasis. However, these mechanisms remain largely unclear. Terminal differentiation might protect cells from apoptosis but the switch for either cell fate is unknown.

We have found a cell-autonomous mechanism that triggers differentiation in response to cell cycle stress in human primary keratinocytes in vitro [4–6]. DNA damage caused by genotoxic agents (chemicals, UV irradiation) [6–8] or oncogenic replication stress, induces squamous differentiation. This differentiation response is triggered via mitotic checkpoints and involves cytokinesis failure and endoreplication. Endoreplication occurs upon G2 or mitotic slippage. Mitotic slippage is the incapability of cell to stay arrested in mitosis upon cell cycle defects [9]. These cells slip from the mitotic checkpoints and enter a new round of DNA replication. As other cell types, keratinocytes can also slip from a G2 arrest, undergoing mitotis bypass [10, 11]. A differentiation-mitosis checkpoint (DMC) would be highly instrumental in developing tissues where apoptosis would be deleterious. However, a DMC or its regulation has not been demonstrated in vivo.

Recent reports have shown DNA damage-differentiation responses in lymphocytes, myeloid cells or neurons [12–14]. Given the potential importance of the DMC to morphogenesis (homoeostasis and regeneration), we have investigated this issue in vivo. To this end, we inactivated Cdk1, Plk1 or CDC20 genes in mouse skin and oral epithelia. The proteins encoded by these genes are required for cell division, and they are checkpoint targets of positive and negative signals at different steps of the G2/M transition prior to cell division. Cdk1 controls mitosis entry from G2 and its inactivation leads to mitosis failure [15, 16]. Plk1 is required for proper chromosomal segregation during mitosis [17, 18]. CDC20 binds to the anaphase-promoting complex (APC) and is required for satisfaction of the spindle assembly checkpoint (SAC) [19, 20].

Inactivation of either Cdk1 or Plk1 produced a similar phenotype, an atrophic epithelium where squamous differentiation was very evident in the basal, normally proliferative layer. Basal differentiation was accompanied with large cells and nuclei, evidence of polyploidy. The tongue lost its villi and instead a premature thickened cornified layer was present. The phenotype was reproduced upon inactivation of AurKB, other regulators of the mitotic checkpoints and also upon overexpression of Plk1 or AurKA, shown to promote polyploidisation [21]. Interestingly, inactivation of CDC20 led to a striking sustained metaphase arrest and induced apoptosis instead of squamous differentiation. Altogether the results demonstrate that the G2/mitotic checkpoints control squamous differentiation. They also provide molecular mechanistic insight into the question why some cells differentiate while others undergo apoptosis in response to DNA damage. We discuss the strong implications into the control of self-renewal tissue homoeostasis and cancer.

## Results

### Inactivation of Cdk1 or Plk1 results in squamous differentiation and polyploidy

Deletion of Cdk1 or Plk1 was generated in vivo. For the Cdk1 line, mice expressing CREerTM (CRE) recombinase under the control of the human Ubiquitin C promoter (UbCRE) [22] or control mice (+) were bred with *Cdk1*lox/lox [23]. For the Plk1 line, mice expressing CREerTM recombinase under the control of the RNA polymerase II locus (PolCRE) [24] were bred with either control (*Plk1*+/+ or *Plk1*+/lox) or *Plk1*lox/lox [18]. CREerTM contains a mutant oestrogen receptor domain rendering Cre activatable by the 4-OH-tamoxifen (TAM) to delete the floxed gene. Two parallel protocols were performed for the induction of the gene knockout (KO). The administration of TAM was systemic (whole body) or topical (local; see “Material and methods”).

The deletion of the floxed sequences in the KO mice upon TAM (Δ) was confirmed by PCR (Supplementary Fig. 1a, b). The lox allele was barely detectable in isolated keratinocytes after treatment with TAM, indicating that the deletion was very efficient. The efficiency of the deletion was also confirmed by immunohistochemical analyses on epidermis in situ (Supplementary Fig. 1c, d). The inactivation of Cdk1 (at a lesser extent) or Plk1 caused DNA damage as monitored by accumulation of the early marker γH2AX (Supplementary Fig. 1e, not shown).

**Fig. 1:**
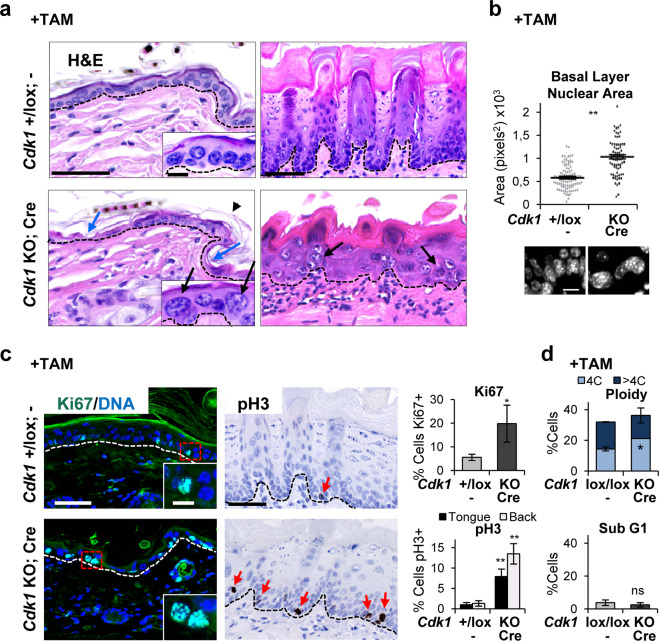
Cdk1 knockout squamous epithelia display perturbed structure and large and polyploid basal nuclei. **a** Representative hematoxylin/eosin (H/E) stain of the epithelium of back skin (left), or the tongue (right) in control mice (*Cdk1*+/lox; −) or KO mice (*Cdk1* KO; Cre) upon TAM (8 days) treatment. Note the loss of cellularity (blue arrows) and the increase in nuclear size (black arrows) in the KO mice. **b** Histogram displaying the basal nuclear area of the epithelium of the tongue of mice as in **a**. Data are mean ± SD of representative immunofluorescence for DAPI (300 nuclei) as in the bottom. **c** Left: representative immunofluorescences for Ki67 of the epidermis (green; DNA in blue by DAPI) of mice as in **a**. Right: representative immunohistochemistry for pH3 of the tongue (brown; DNA in blue by hematoxylin) of mice as in **a**. Note the single pH3 positive cell in the control compared with the frequent positive cells in the KO epidermis (red arrows). Bar histograms: percent of Ki67 (top) or pH3 (bottom) positive cells. Data are mean ± SD of five representative fields (more than 500 nuclei). **d** Bar histograms for the percent of 4C (G2/M + tetraploids), >4C (polyploid), or sub-G1 cells (as measured by propidium iodide, PI) isolated from control mice (*Cdk1*lox/lox; −) or KO mice (*Cdk1* KO; Cre) upon TAM (7 days) treatment. Broken line for the basement membrane. Scale bars: **a**, **c** 50 μm, **d** 10 μm; inset scale bar **a**, **c** 10 μm. **P < 0.01, *P < 0.05, ns not significant. Control mice do not have CRE recombinase (−). The results are representative of 2–3 animals per group (*N* = 2–3) and two independent experiments. See also Supplementary Fig. 2.

The results of the histological analyses by hematoxilin/eosin (H/E) of the skin and oral mucosa of Cdk1 or Plk1 KO mice after TAM treatment were very similar. Cdk1 KO epidermis was thinner than wild type (Fig. 1a, left). In addition, KO epidermis was hyperkeratotic with thickened cornified layer (arrowhead). There was also a striking loss of cellularity and patches of cornified cells in the basal layer (Fig. 1a, blue arrows). Interestingly, the nuclei of the basal, normally proliferative layer, of KO skin were larger than controls (Fig. 1a, black arrows; Supplementary Fig. 2a). The oral epithelium, generally more hyperplastic than the epidermis, was unstructured and villi of the tongue were small and distorted (Fig. 1a, right). The morphology of the proliferative basal layer was lost and instead giant cells and nuclei were found (Fig. 1a, b).

Frequent mitotic abnormalities were identified in the skin of Cdk1 KO mice after TAM treatment. The expression of the proliferative marker Ki67 was increased (Fig. 1c). Cells expressing pH3, a specific marker of mitotic chromosome condensation, in the oral mucosa of the tongue (Fig. 1c) or epidermis (not shown) were very frequent, suggesting a prolonged mitotic arrest. The accumulation of both Ki67 and pH3 in the Cdk1 KO epithelia suggests that cells arrest in the G2 to mitosis transition. Ki67 positive cells were larger in Cdk1 KO epidermis in comparison with the wild-type mice (Fig. 1c, Supplementary Fig. 2a), further supporting a G2/M arrest, when nuclei double their size by DNA replication. Consistently, the evaluation of DNA content by propidium iodide (PI) showed a significant increase of the tetraploid (4C) population of keratinocytes (Fig. 1d). No significant apoptosis was detected in KO cells according to sub-G1 DNA content (Fig. 1d).

**Fig. 2:**
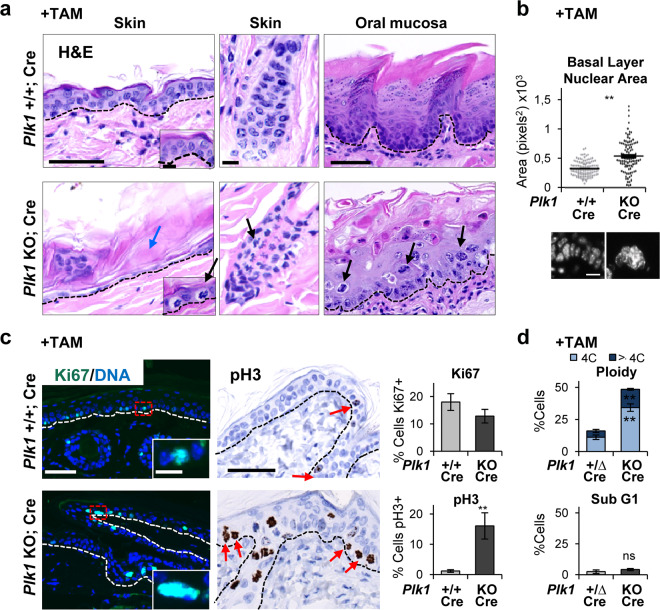
Plk1 knockout squamous epithelia display striking mitotic accumulation, polyploidy and dyskeratosis. **a** Representative H/E stain of the epithelium of back skin (left and middle), or the tongue (right) in control mice (*Plk1*+/+; Cre) or KO mice (*Plk1* KO; Cre) upon TAM (6 days) treatment. Note the loss of cellularity (blue arrows) and the numerous metaphases (black arrows) in the KO epidermis. **b** Histogram displaying the basal nuclear area of the epithelium of the tongue of mice as in **a**. Data are mean ± SD of representative immunofluorescence for DAPI (300 nuclei) as in the bottom. **c** Left: representative immunofluorescences for Ki67 (as in **a** and as Fig. 1). Right: representative immunohistochemistry for pH3 of the epidermis of mice as in **a** 4 days after TAM treatment (brown; DNA in blue by hematoxylin). Note the very frequent pH3 positive cells in the KO epithelia (red arrows). Bar histograms: corresponding percent of Ki67 or pH3 positive cells, as indicated. Data are mean ± SD of five representative fields (more than 500 nuclei). **d** Bar histograms for the percent of 4C (G2/M + tetraploids), >4C (polyploid) or sub-G1 cells (as measured by PI) isolated from control mice (*Plk1*+/∆; Cre) or KO mice (*Plk1* KO; Cre) upon TAM (7 days) treatment. Broken line for the basement membrane. Scale bars: (**a**-left, **a**-right, **c**): 50 μm, (**a**-middle, **d**): 10 μm; inset scale bar **a**, **c** 10 μm. **P < 0.01, ns not significant. The deletion of the floxed sequences is represented by Δ. The results are representative of 2–3 animals per group (*N* = 2–3) and three independent experiments. See also Supplementary Fig. 2.

Stratified epithelia of Plk1 KO mice displayed a similar phenotype to the Cdk1 KO mice described above. Thin epidermis, parakeratosis, differentiated basal layer (dyskeratosis), distorted oral epithelium (Fig. 2a). Basal nuclei were also large both in epidermis (Supplementary Fig. 2a-bottom) and in oral mucosa (Fig. 2b). Increased frequency of mitotic pH3 positive cells (Fig. 2c) and frequent abnormal mitoses (Fig. 2a, black arrows) were also found. The main difference with Cdk1 epithelium was that Ki67 did not accumulate, possibly due to the later function of Plk1 within G2/M (Fig. 2c; [17]). However, and consistently wit Cdk1 KO epidermis, Ki67 positive cells were larger in Plk1 KO epidermis (Supplementary Fig. 2a-bottom). As observed in Cdk1 KO cells, the tetraploid (4C) and polyploid (>4C) populations increased in Plk1 KO epithelia (Fig. 2d), whereas no significant sub-G1 apoptosis was detected (Fig. 2d).

**Fig. 3:**
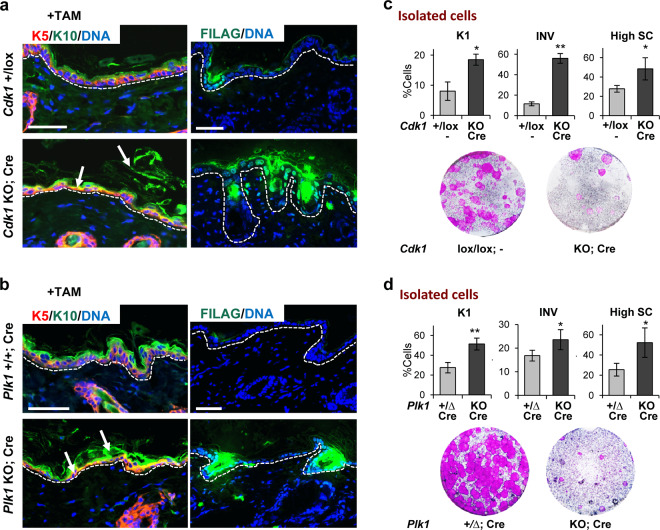
The loss of Cdk1 or Plk1 results in premature basal terminal differentiation. **a** Representative double immunofluorescence of back epidermis of control mice (*Cdk1*+/lox;−) or KO mice (*Cdk1* KO; Cre) upon TAM (8 days) treatment for squamous markers keratin K5 (red) and keratin K10 (green) or filaggrin (FILAG, green). DNA in blue (DAPI). Broken line for the basement membrane. Scale bars: 50 μm. **b** Representative double immunofluorescence as in **a** of back epidermis of control mice (*Plk1*+/+; Cre) or KO mice (*Plk1* KO; Cre) upon TAM (6 days) treatment. **c** Bar histograms display the percent of involucrin (INV) positive cells, keratin K1 positive cells or cells with high scatter (High SC) from mice in **a** measured by flow cytometry. Bottom: representative clonogenic capacity of cells from mice as in **a**. **d** Bar histograms display the percent of involucrin (INV) positive cells, keratin K1 positive cells or cells with high scatter (High SC) from mice as in **b** 7 days after treatment measured by flow cytometry. Bottom: representative clonogenic capacity of cells as in **b** 7 days after treatment measured. **P < 0.01 *P < 0.05. Cdk1 control mice do not have CRE recombinase (−). The deletion of the floxed sequences is represented by Δ. Data are mean ± SD of 2–3 animals per group (*N* = 2–3) and three independent experiments. See also Supplementary Fig. 2.

Double labelling of the skin for differentiation markers further confirmed that the stratified epithelia were terminally differentiated in Cdk1 and Plk1 KO mice. Proliferative basal keratin K5 (red) and postmitotic terminal differentiation marker keratin K10 (green) revealed frequent large patches of terminally differentiated cells in the basal layer coexpressing both markers (Fig. 3a, b). These observations indicate that basal cells in the absence of Cdk1 or Plk1 undergo premature squamous terminal differentiation. We also analysed late differentiation markers filaggrin and involucrin. They were upregulated in Cdk1 or Plk1 KO epidermis upon TAM (Fig. 3a, b; Supplementary Fig. 2b). The induction of differentiation in the basal layer was also confirmed by double labelling for proliferative basal K5 (red) and the oral-specific differentiation marker keratin K13 (green) in oral epithelia (Supplementary Fig. 2b).

Keratinocytes isolated after gene inactivation were analysed by flow cytometry (FC) (Fig. 3c, d). Expression of differentiation markers K1 or involucrin was significantly increased compared with controls. In addition, cells were larger and more complex (high forward and side light scatter parameters; High SC), typical changes of squamous differentiation [25]. Confirming the generalised induction of irreversible terminal differentiation, keratinocytes from KO epidermis were unable to proliferate as demonstrated by clonogenicity assays in the absence of TAM (Fig. 3c, d).

**Fig. 4:**
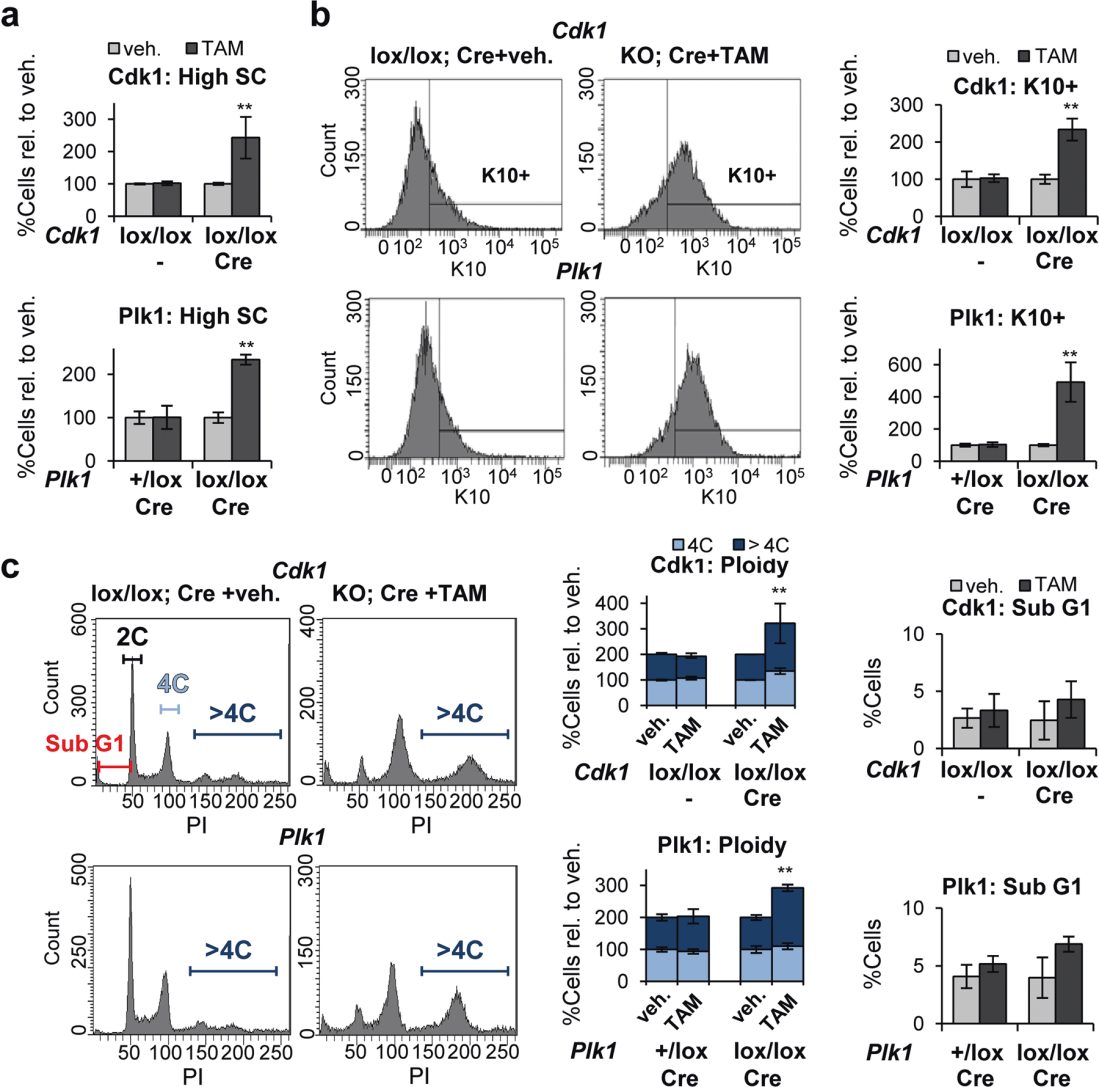
Inactivation of Cdk1 or Plk1 in freshly isolated cells induces squamous differentiation. **a** Percent of cells with high-scatter parameters (high SC), in Cdk1 control cells (*Cdk1*lox/lox;−) or Cdk1lox-cre cells (*Cdk1*lox/lox; Cre) 3 days after treatment with TAM or with vehicle (veh.) only (top) or, Plk1 control cells (*Plk1*+/lox; Cre) or Plk1lox-cre cells (*Plk1*lox/lox; Cre) 4 days after treatment with TAM or with veh only (bottom), relative to keratinocytes treated with veh. **b** Representative flow-cytometry histograms for the differentiation marker keratin K10 (K10+, positive cells according to negative isotype antibody control, not shown). Bar histograms show the percent of K10 positive cells as in **a** relative to keratinocytes treated with veh. **c** Representative flow-cytometry analyses of DNA content. Bar histograms: percent of 4C cells (G2/M + tetraploids), >4C cells (Polyploid) or in the Sub-G1 fraction of the cell cycle (as measured by PI), as in **a** and relative to keratinocytes treated with veh. **P < 0.01. Cdk1 control mice do not have CRE recombinase (−). Data are mean ± SD of duplicate or triplicate samples (*n* = 2 or *n* = 3) of at least two mice per group (*N* = 2) and three independent experiments. See also Supplementary Figs. 3, 4.

Isolating differentiating keratinocytes from the skin is hard due to the difficult access of trypsin into the differentiated layers. To circumvent this limitation, we induced inactivation of Cdk1 or Plk1 in vitro. We isolated cells from non-treated mice, placed them in culture and added TAM for 3–4 days. The changes observed confirmed those obtained upon TAM in vivo. The clonogenic capacity of Cdk1 or Plk1 KO keratinocytes was again impaired (Supplementary Figs. 3a and 4a). KO cells displayed high SC (Fig. 4a, Supplementary Figs. 3b and 4b) and increased population of cells expressing differentiation markers K10 (Fig. 4b, Supplementary Figs. 3c and 4c) or involucrin (Supplementary Figs. 3d and 4d). KO keratinocytes strikingly accumulated in G2/M (4C) and polyploidy (>4C; Fig. 4c, Supplementary Figs. 3e and 4e), in a similar fashion in both Cdk1 and Plk1 KO mice. Only a small proportion of cells were found in apoptotic sub-G1 (Fig. 4c).

The results on the cell cycle and differentiation of Cdk1 and Plk1 KO cells in vitro were more striking than those obtained in vivo. This might be due to the difficult extraction of differentiated cells from the epidermis, but also to a slower turnover in vivo, considering that in vitro conditions are cell cycle stimulatory. We induced hyperproliferative conditions in vivo by topical treatment with 12-O-tetradecanoylphorbol-13-acetate (TPA), a well-known activator of epidermal proliferation [26]. This was combined with addition of TAM for deletion of *Plk1*. Interestingly, Plk1 KO epidermis treated with TPA contained very frequent mitotic figures by 7 days and was acutely hyperkeratotic (cornified) by 10 days, with loss of the undifferentiated layers (Supplementary Fig. 5). Plk1 KO tongue epithelium upon TPA also appeared grossly perturbed and differentiated displaying frequent suprabasal mitotic figures (Supplementary Fig. 5).

Although we have studied the squamous epithelia of mice KO for Cdk1 or Plk1 more in depth, we have observed a similar epidermal phenotype in mice KO for mitotic AurKB or AurKA and in transgenic mice overexpressing AurKA or Plk1 (Supplementary Fig. 6 and not shown).

### Cytokinesis impairment triggers polyploidy differentiation

Altogether the results show that mitosis failure in keratinocytes leads to squamous differentiation involving polyploidy (endoreplication). The mechanism driving this response is unclear, since the loss of Cdk1 or Plk1 is thought to produce cell cycle arrest in different phases of G2/M. Consistently, as described above, Cdk1 KO cells displayed large single nuclei typically polyploid (Supplementary Fig. 3f), in contrast to Plk1 KO cells that were frequently binucleated or multinucleated (Supplementary Fig. 4f), suggesting that they are capable of undergoing nuclear division. The fact that inactivation of either gene produced similar effects, suggests that cytokinesis failure due to mitotic defects might trigger differentiation. To test this, we treated freshly isolated human epidermal keratinocytes with drugs that impair cytokinesis, Blebbistatin or Cytochalasin D. Blebbistatin is a specific myosin II inhibitor [27] and Cytochalasin D at low concentration induces tetraploidy by inhibiting actin filaments [28].

**Fig. 5:**
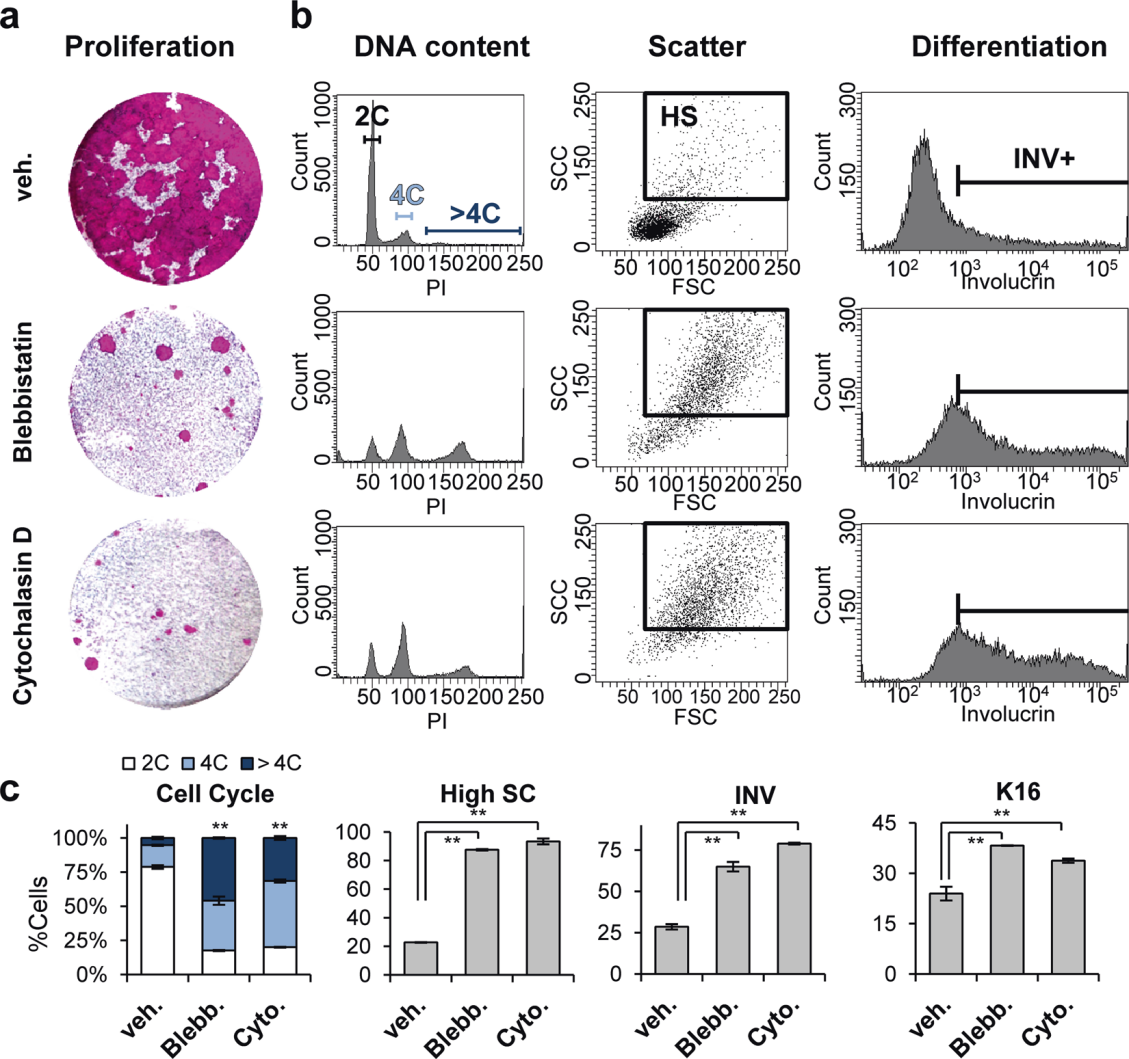
Inhibition of cytokinesis in human epidermal cells triggers squamous differentiation. Primary cells from human epidermis were treated with vehicle (veh.), blebbistatin (Blebb.) or cytochalasin (Cyto.) for 48 h. **a** Clonogenic capacity of cells drug-released and plated (3000 cells per well in triplicate samples) after treatment as indicated. **b** Representative flow-cytometry analyses for DNA content, morphology (light scattering) and expression of the differentiation marker involucrin (INV+, positive cells), as indicated. **c** Bar histograms: percent of 2C (G1, dyploid), 4C (G2/M + tetraploids) or >4C (polyploid) cells as measured by PI; percent of cells with high light scatter features (high SC); percent of INV+ cells; or percent of keratin K16 positive cells, as indicated. **P < 0.01. Data are mean ± SD of triplicate samples (*n* = 3) and three independent experiments. See also Supplementary Fig. 4.

Keratinocytes treated with either Blebbistatin or Cytochalasin D for 48 h lost the ability to divide as expected (Fig. 5a) and accumulated in the 4C/polyploid fractions of the cell cycle (Fig. 5b, c). Binucleate cells were frequent (Supplementary Fig. 4g). Interestingly, cytokinesis impairment led to an increase in cell size and complexity (high SC) and a sharp induction of squamous suprabasal differentiation markers, such as involucrin (Fig. 5b, c) or keratin K16 (Fig. 5c).

### CDC20 is required for differentiation

The results above show that keratinocytes undergo differentiation when cell division fails by different means, G2/mitotic slippage or cytokinesis failure. However, it is unclear whether keratinocytes need to progress into cytokinesis upon mitosis failure in order to initiate squamous differentiation. To address this relevant question, we inactivated CDC20 as we did for Cdk1 or Plk1. CDC20 is an essential cofactor of the anaphase-promoting factor (APC) and is required for anaphase once the spindle assembly checkpoint (SAC) is satisfied [19]. In the absence of CDC20 cells cannot exit mitosis [20].

Mice expressing PolCRE [24] or control mice (+) were bred with either control (*CDC20*+/+) or *CDC20*lox/lox [20] mice. The efficiency of the lox deletion upon TAM was confirmed by epidermal DNA isolation and diagnostic PCR (Supplementary Fig. 7a). CDC20 KO cells were unable to proliferate (Supplementary Fig. 7b).

**Fig. 6:**
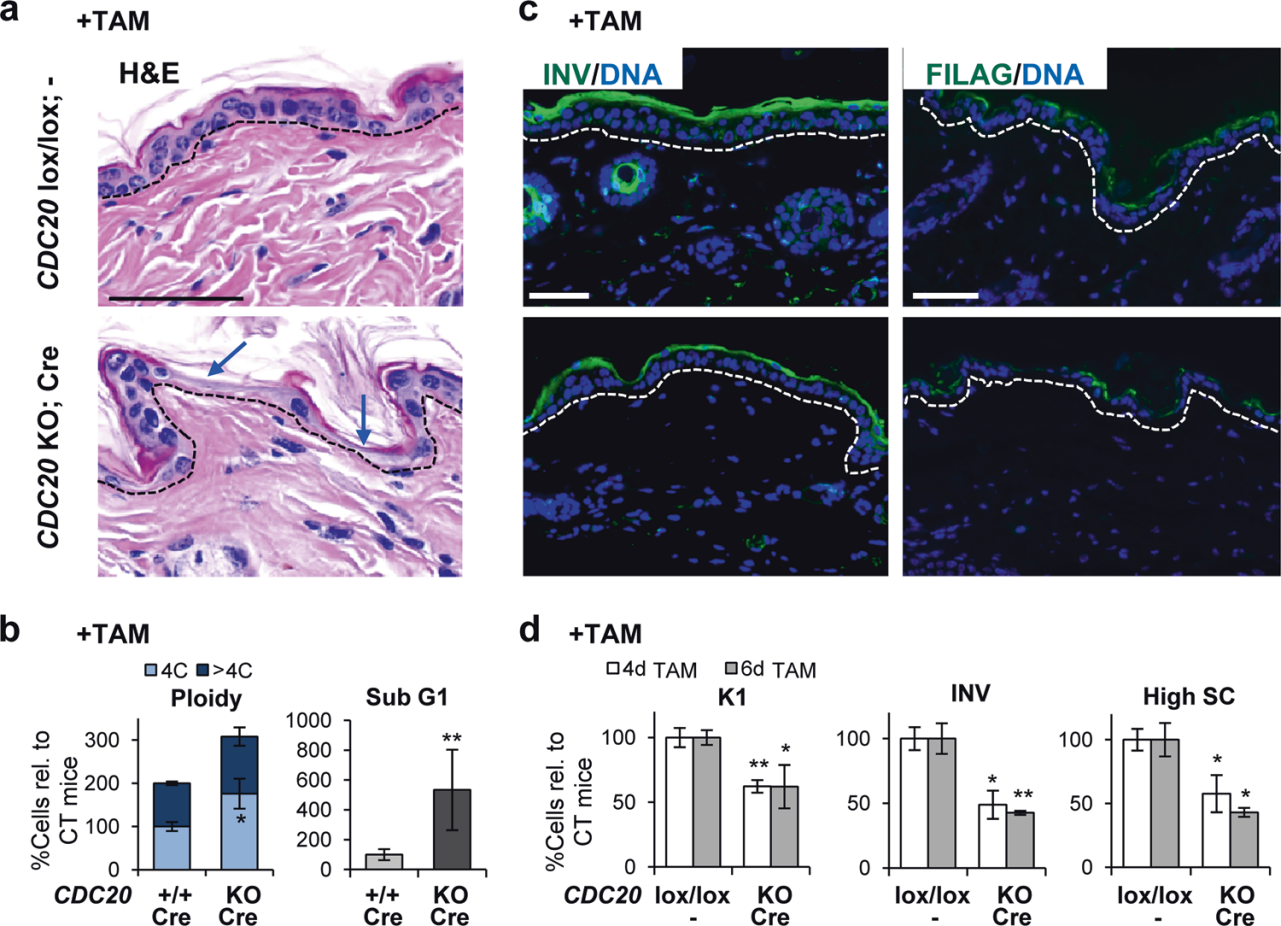
CDC20 is required for terminal squamous differentiation. **a** Representative H/E stain of the skin of control mice (*CDC20*lox/lox; −) or KO mice (*CDC20* KO; Cre) upon TAM (6 days) treatment. Note the striking loss of cellularity (blue arrows). **b** Bar histograms display the percent of cells 4C (G2/M + tetraploids), polyploid (>4C) or in the sub-G1 fraction of the cell cycle (as measured by PI), all relative to CT mice, in control mice (*CDC20*+/+; Cre) or KO mice (*CDC20* KO; Cre) upon TAM (7 days) treatment. **c** Representative immunofluorescences of squamous differentiation markers: involucrin (INV, green) or filaggrin (FILAG, green) of epidermis of mice as in **a** DNA in blue (DAPI). **d** Bar histograms: percent of keratin K1 positive cells, INV positive cells or high-scatter cells (high SC) measured by flow cytometry, relative to CT mice, after 4 or 6 days treatment in mice as in **a**. Broken line for the basement membrane. Scale bars: 50 μm. **P < 0.01 *P < 0.05. CT: *CDC20*lox/lox; − or *CDC20*+/+; Cre mice. Data are mean ± SD of 2–3 animals per group (*N* = 2–3) and three independent experiments. See also Supplementary Fig. 7.

Consistently with Cdk1 or Plk1 KO epidermis, there was a remarkable loss of cellularity in the basal layer (Fig. 6a). However, in this case the nuclei of CDC20 KO basal keratinocytes were smaller than controls (Supplementary Fig. 7c). Mitotic figures were abnormally found all throughout the epidermis of CDC20 KO mice upon systemic TAM treatment. This was further monitored by detection of pH3 (Supplementary Fig. 7d). Consistently, FC analyses of freshly isolated keratinocytes showed an increase in the 4C population (Fig. 6b) while there were no significant changes in the polyploid fraction of the cell cycle (>4C). However, the sub-G1 apoptotic population was markedly increased (Fig. 6b). Proliferative Ki67 positive cells were scarce (Supplementary Fig. 7d).

Inactivation of CDC20 was not accompanied by an increase in differentiation markers: involucrin (Fig. 6c, d; Supplementary Fig. 7e), filaggrin (Fig. 6c) or keratin K10 (Supplementary Fig. 7e, f) or K1 (Fig. 6d) were downregulated in epidermis. Furthermore, freshly isolated keratinocytes displayed a reduction in cellular size and complexity, 4 and 6 days upon TAM treatment (High SC, Fig. 6d). These changes are opposite to those induced by squamous differentiation. Analyses of oral epithelium also showed frequent mitotic figures and loss of the differentiation marker keratin K13 (Supplementary Fig. 7g).

**Fig. 7:**
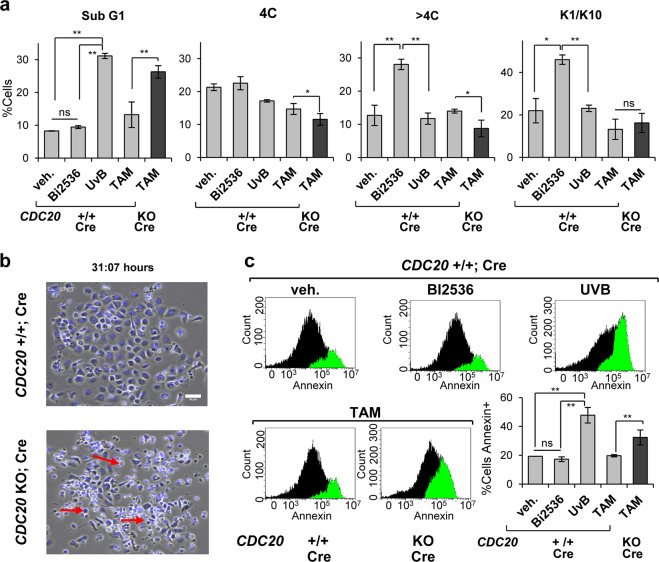
Isolated CDC20 KO epithelial cells undergo apoptosis. **a** Apoptotic sub-G1 fraction as measured by PI, in control cells (*CDC20*+/+; Cre), upon vehicle only (48 h), Plk1 inhibitor BI2536 (48 h), lethal UV irradiation (48 h) or TAM for 3 days, as indicated; black bar for *CDC20* KO; Cre cells upon TAM treatment for 3 days. Percent of cells 4C (G2/M + tetraploids) and >4C (polyploids) 48 h after BI2536 or UV irradiation, or 4 days after TAM. Fraction of cells positive for squamous differentiation markers K1 and K10 48 h after BI2536 or UV irradiation, or 3 days after TAM. **c** Frame from a representative time-lapse video by phase-contrast microscopy and DNA labelling by NucBlue, 31 h after addition of TAM to delete *CDC20*. Scale bar: 50 μm. **b** Flow-cytometry analyses for detection of apoptotic early marker Annexin V after BI2536 (48 h), UV irradiation (48 h) or TAM (4 days). Bar histogram displays quantification of Annexin positive cells. **P < 0.01, *P < 0.05, ns not significant. Data are mean ± SD of duplicate or triplicate samples (*n* = 2 or *n* = 3) of at least two mice per group (*N* = 2) and five independent experiments. See also Supplementary Fig. 8.

To monitor the effects of CDC20 inactivation, we induced the deletion in freshly isolated cells in vitro. Whereas Plk1 chemical inhibitor (BI2536) had no effect on sub-G1, analyses of DNA content systematically showed a striking population of apoptotic CDC20 KO cells in sub-G1 (Fig. 7a, Supplementary Fig. 8a), at levels comparable with acute UVB irradiation (Fig. 7a) [7]. There was also loss of the G2/M (4C) and polyploid (>4C) fractions (Fig. 7a). Deletion of CDC20 did not induce differentiation but apoptosis as monitored by the differentiation markers K1 and K10 (Fig. 7a) or life cell analysis (Fig. 7b). To further confirm that the inactivation of CDC20 caused apoptosis, we made use of the early apoptotic marker Annexin V. As shown in Fig. 7c, whereas cells were unaffected by treatment with Plk1 inhibitor, acute UVB irradiation or deletion of CDC20 by TAM induced a striking increase in the expression of Annexin V.

**Fig. 8:**
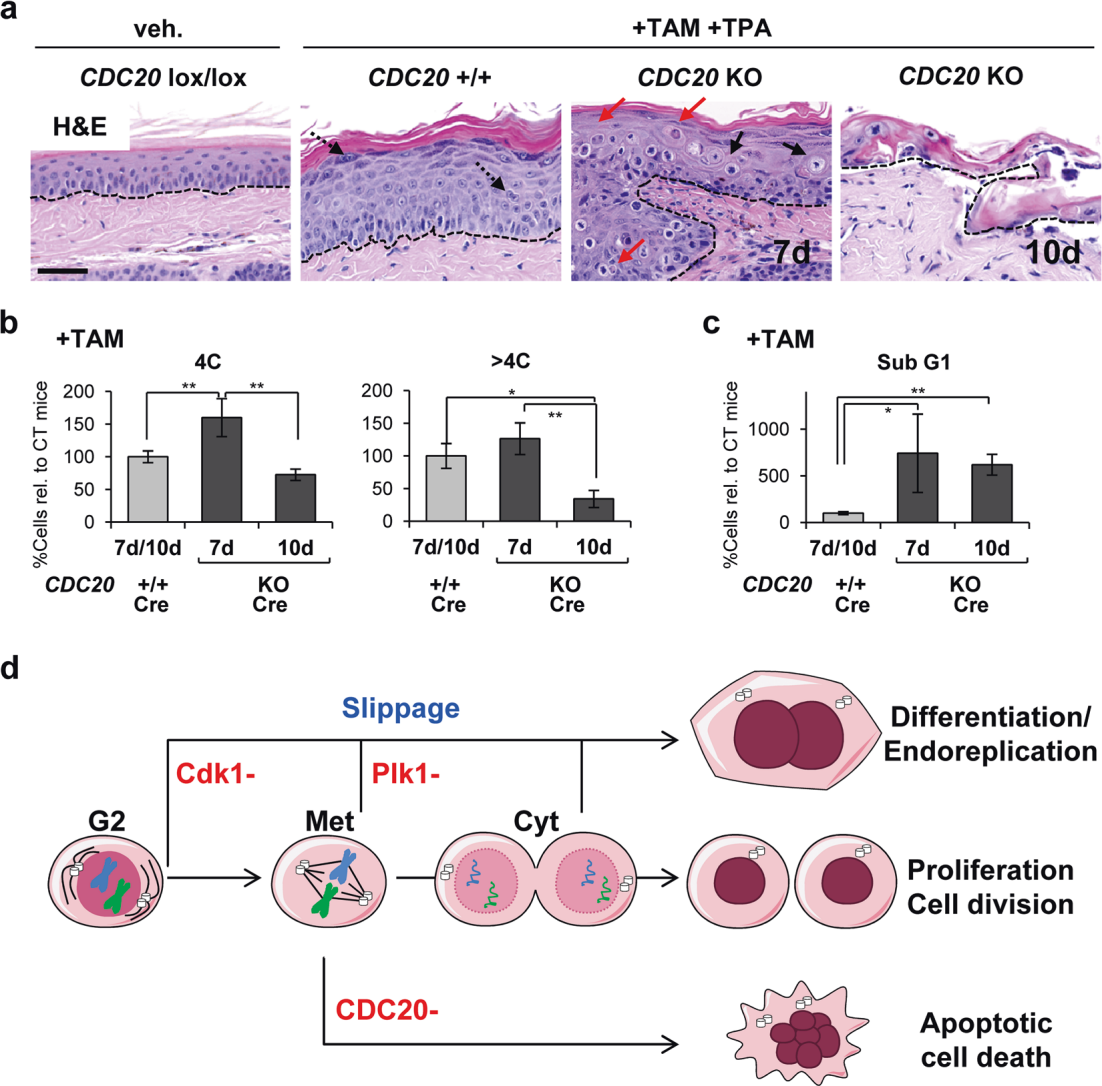
Inactivation of CDC20 drives epidermal hyperplasia into cell death. **a** H/E stain of epidermis of the tail of control (*CDC20*+/+; Cre) or KO mice (CDC20 KO; Cre) after 7 or 10 days upon topical treatment with vehicle only or TAM and TPA, as indicated. Note abnormal metaphases (black arrows) and apoptotic bodies (red arrows) in the KO mice. Broken line for the basement membrane. Scale bars: 50 μm. **b** Percent of 4C (G2/M + tetraploids) or >4C (polyploid) or **c** apoptotic sub-G1 cells directly isolated from epithelia in **a**, as indicated. **P < 0.01 *P < 0.05. Data are mean ± SD of three animals per group (*N* = 3) and two independent experiments. See also Supplementary Fig. 8. **d** Model for the control of squamous differentiation by the mitotic checkpoint molecules studied. Depletion Cdk1 or Plk1 results in mitotic slippage and endoreplication and differentiation. However, inhibition of mitotic slippage by complete inactivation of CDC20 impairs squamous differentiation and results in apoptosis. Met metaphase; Cyt cytokinesis.

We then stimulated the epidermis of the tail to undergo hyperproliferation in vivo by 7 or 10 days TPA and TAM treatment. CDC20 KO epidermis 7 days after TPA and TAM treatment displayed a great frequency of apoptotic figures all through the epithelium (Fig. 8a). By 10 days the epidermis of the tail was mostly detached. The remaining tissue contained no living epidermis (Fig. 8a). Cell isolates displayed an increase of the mitotic 4C population by 7 days (Fig. 8b). However, by 10 days mitotic (4C) and polyploid (>4C) fractions diminished (Fig. 8b). To note, mitotic arrest in 4C was accompanied by a higher ratio of early apoptosis (sub-G1, Fig. 8c) and the presence of numerous mitotic figures even in very suprabasal layers, both in epidermis and oral epithelium (Fig. 8a, Supplementary Fig. 8b, c). Therefore, the results consistently show that CDC20 KO keratinocytes undergo apoptosis. Although the cell fate differed from Cdk1 or Plk1 KO, CDC20 KO epithelia displayed strongly positive γH2AX cells throughout the tissue (Supplementary Fig. 8d).

To analyse differentiation, we performed double immunostaining for keratins K5 and K10 or single immunostaining for involucrin. In contrast to what observed on Cdk1 (not shown) or Plk1 KO epidermis, both markers showed no increase of terminal differentiation by 7 days and only some remains of an aberrant cornified layer were found by 10 days (Supplementary Fig. 8b). The loss of CDC20, but not that of Cdk1 or Plk1, caused a striking reduction of PCNA positive cells (Supplementary Fig. 9a, b). PCNA is a marker of cell cycle progression and DNA replication [29]. Sustained cell cycle in Cdk1 or Plk1 KO cells is consistent with G2/M slippage and endoreplication, whereas the decrease in cell cycle activity in CDC20 KO is consistent with a permanent mitotic arrest. PCNA was also often detected in differentiating K10 or involucrin-positive cells (Supplementary Fig. 9a, b). We also found frequent apoptotic TUNEL positive cells in intermediate layers of the CDC20 KO squamous epithelia of TPA treated animals (Supplementary Fig. 9c).

Altogether the results consistently show that in the absence of CDC20 keratinocytes is not driven into squamous differentiation but into apoptosis. Interestingly, endogenous CDC20 strongly accumulates in mitotic stratifying cells of human skin (Supplementary Fig. 10; [30]), further suggesting that it is required for the initiation of squamous differentiation from mitosis arrest.

## Discussion

DNA damage links oncogenic alterations with antiproliferative signals due to replication stress [31]. Replication stress triggers checkpoints of G2 or mitosis [32]. By directly causing mitotic stress at different levels we demonstrate the existence of a differentiation mitosis checkpoint (DMC) in squamous epithelia of the skin and oral mucosa.

By comparing the epithelia in the absence of Cdk1, Plk1 or CDC20 we now can begin to elucidate the molecular regulation of keratinocyte responses. Keratinocytes are particularly resistant to apoptosis unless acute exposure to UV irradiation (sunburn) [33, 34], and they undergo differentiation upon stimuli that are apoptotic in other cell types [35]. The reasons for this keratinocyte resistance to apoptosis are unclear. In addition, differentiating keratinocytes undergo endoreplication and become polyploid [8, 36]. It has been suggested that endoreplication might protect differentiating cells from apoptosis [7, 37–40]. A differentiation response to DNA damage might be essential in tissues exposed to genetic insult that fulfil a specialised function, where apoptosis would be deleterious. It is interesting that deregulation of the keratinocyte cell cycle by overexpression of MYC, Cyclin E or inactivation of p53 causes DNA damage, mitotic slippage and terminal differentiation [5, 6, 8]. Our results now provide mechanisms for the prevailing keratinocyte differentiation fate in response to genetic damage.

Cdk1 is essential for cells to accomplish metaphase and cell division [41, 42]. In the absence of Cdk1 cells arrest in the G2/M boundary [15, 16, 43]. The complex Cyclin B1/Cdk1 must be active up to metaphase. It has been shown that mitotic slippage results from premature inactivation of Cdk1 during G2 or mitosis arrest [44–46]. In this case, cells undergo cytokinesis prematurely failing to cell division and entering a special S phase and DNA replication. Cdk1 KO epithelia displayed mitotic figures and cells expressing the mitotic marker pH3 involved in chromatin condensation [47]. Due to the endoreplication response Cdk1 KO keratinocytes continued DNA replication and became polyploid. Mitosis impairment in proliferative basal cells not only provoked atrophy, also it produced a gross alteration of tissue structure, with the basal cells resembling typical superficial layers. The reason for this phenotype was rapid differentiation of basal cells. Similar results were obtained in situ and in vitro in the absence of Plk1 in spite of acute DNA damage. The loss of Plk1 function allows cells to enter mitosis, but leads to defects in chromosome segregation, mitotic slippage and cytokinesis failure [21, 48]. We found a similar epidermal phenotype in the squamous epithelia of mice lacking mitosis kinase AurKB, also required for proper chromosomal segregation and in mice overexpressing AurKA or Plk1 that also display increase in ploidy due to cytokinesis impairment [21]. We conclude that deficient mitotic exit involving mitotic slippage and impairment of cytokinesis in vivo leads to squamous differentiation.

Endoreplication allows postmitotic cells to become larger and it is typical of differentiating cells that need to significantly increase their cellular mass and volume [11, 49, 50]. Plant epidermal or Drosophila epithelial cells are well known for becoming large due to endoreplication. Similarly, in human an increasing variety of cell types are currently known to become large by endoreplication [10]. For instance, suprabasal keratinocytes enlarge as they differentiate [34, 51], megakaryocytes fragment into platelets after polyploidysation [52], and mammary milk producing cells became large and binucleated [53]. Therefore, it is legitimate to question whether in these systems differentiation requires G2/mitotic slippage and endoreplication. Our results after deletion of CDC20 in vivo provide insight into this issue. In the absence of CDC20 the APC cannot be activated, chromosomes stay attached to the kinetochores and therefore cells cannot undergo G2/mitotic slippage or endoreplication. In the absence of CDC20, keratinocytes were stuck in mitosis and accumulated DNA damage. However, instead of differentiation they underwent apoptosis both in situ and in vitro. Therefore, keratinocytes must slip beyond mitosis in order to differentiate. Keratinocytes differentiate upon stimuli that in other cell types induce apoptosis, such as lack of anchorage [54], DNA damage, oncogene activation [4] or mitotic stress. Our results suggest that the cell fate switch lies in the G2/mitotic checkpoints. The capacity of keratinocytes to undergo G2/mitotic slippage protects them from apoptosis. It is simple and elegant of a differentiating self-renewal tissue to link cell cycle and genomic defects with the enlargement and protein production needed for fulfilling its function.

Altogether the results in vivo and on freshly isolated cells in vitro showed that cytokinesis failure drives squamous differentiation. Although a cytokinesis checkpoint is not well established, some evidence supports its existence [55, 56]. This checkpoint might determine the final cellular choice towards cell division or differentiation. The fact that in our assays the inhibition of cytokinesisinduced squamous differentiation supports this hypothesis.

The question now is why other cell types undergo apoptosis in response to cell cycle stress, acute DNA damage and mitotic failure (mitotic catastrophe) [2, 3] while keratinocytes undergo squamous differentiation. Keratinocytes have a limiting mitotic Cdk1, possibly due to low-expression levels of the activating ligand Cyclin B, or to the high-transient expression of the cdk inhibitor p21 [8, 57, 58]. As discussed above, there is solid evidence that mitotic slippage results from inactivation of Cdk1 during G2/mitotic arrest. Partial inhibition of Cdk1 in G2 phase overrides the SAC and decouples mitotic events. Interestingly, inhibition of p21 by AurKB suppresses premature exit of mitosis [59]. Therefore, in keratinocytes inactivation of Cdk1 during G2 or mitosis arrest might be required to allow G2/M slippage and to drive irreversible terminal differentiation. Within these lines, it is interesting that knocking-down Cdk1 inhibitor Wee1 drives UV-induced keratinocyte differentiation into apoptosis [7]. Consistently, CDC20 strongly accumulates in mitotic cells in differentiating layers of the epidermis. Keratinocytes seem to require CDC20 in order to inhibit Cdk1, loosen G2/M arrest, avoid apoptosis and trigger squamous differentiation (Fig. 8d). The mechanisms by which polyploidy protects cells from apoptosis are unclear. However, accumulation of CDC20 by activating the APC and allowing mitosis exit in spite of cell cycle defects might suppress cytokinesis and promote terminal differentiation. This would in turn suppress apoptosis. Conversely, a more robust sustained G2/M arrest might drive other cells into apoptosis. Within these lines, mitotic slippage might be required for terminal differentiation to avoid apoptosis and subsequently, to allow the protein mass production and the significant cellular growth that are necessary for the making of the cornified layer of the skin [51]. The implications of a differentiation DNA damage response into the control of squamous homoeostasis are important. Squamous epithelia continuously undergo self-renewal, and they are continuously exposed to genetic injury. They need rapid mechanisms suppressing proliferation of cells that are no longer reparable whose multiplication would lead to genomic instability. It is interesting that benign squamous hyperplasia involves paradoxical thickening of the differentiated component. Consistently, in our studies hyperplasia-induced TPA led to a higher ratio of differentiation in the absence of Cdk1 or Plk1 (to a higher ratio of apoptosis in the absence of CDC20).

In summary, whether sustained mitotic arrest drives apoptosis or differentiation in response to DNA damage seems to be cell type dependent and to respond to a different regulation of mitotic checkpoints. Although DNA damage-induced apoptosis has been more thoroughly studied, an increasing body of human tissues is being shown to respond to genetic damage with endoreplication or polyploidisation [10]. Dissecting the cellular mechanisms leading to either apoptosis or differentiation is essential to understand the control of tissue homoeostasis.

## Material and methods

### Animal models

All animal procedures were approved by the Comité de Ética de la Investigación y de Bienestar Animal of the Instituto de Salud Carlos III and Comunidad de Madrid, Spain and by the Ethical Committee of the Consejería de Ganadería, Pesca y Desarrollo Rural of the Gobierno de Cantabria (PI-15-17).

Mice were housed at the pathogen-free animal facility of the Centro Nacional de Investigaciones Oncológicas (Madrid) following the animal care standards of the institution. They were delivered to Servicio de Estabulación y Experimentación Animal of the Universidad de Cantabria. These animals were observed on a daily basis, and sick mice were euthanised in accordance with the Guidelines for Humane End Points for Animals used in biomedical research. Male and female mice 6–12 weeks after birth were used.

The conditional mice were previously generated. In lox mice: in *Cdk1*lox mice, the murine *Cdk1* exon 3 was flanked with loxP sites [23]; in *Plk1*lox mice, the murine *Plk1* exon 2 was flanked with loxP sites [18]; in *CDC20*lox mice, the *CDC20* exon 2 was flanked with loxP sites [20]; in *AuKB*-lox mice, the *AurKB* exons 2–6 were flanked with loxP sites [60].

To conditionally generate a null allele of each gen we made use of two commonly utilised whole-body TAM-inducible Cre recombinase strains: a transgenic line expressing the TAM-inducible Cre-ERT2 recombinase [61] under the control of the human UbCRE [22] or a knock-in strain expressing the inducible Cre-ERT2 recombinase under the control of the locus encoding the large subunit of RNA polymerase II (PolCRE) [24]. UbCre(−) or UbCRE(Cre) mice were crossed with *Cdk1*lox/lox or *Cdk1*+/lox, or *AurKB*lox/lox mice. PolCRE(Cre) mice were crossed with *Plk1*+/+, *Plk1*+/lox or *Plk1*lox/lox mice. PolCRE(−) or PolCRE(Cre) mice were crossed with *CDC20*+/+, *CDC20*+/lox or *CDC20*lox/lox mice. Genotyping was performed as previously described for each strain used.

In knockin mice: in *Plk1*+/KI a cassette containing the FLAG-human *Plk1* cDNA under the control of the doxycycline (Dox)-responsive promoter (tetO) was inserted downstream of the Col1A1 locus [21]; in *AurKA*+/KI a cassette containing the FLAG-human *AurKA* cDNA under the control of the Dox-responsive promoter (tetO) was inserted downstream of the Col1A1 locus [62]. To conditionally generate a KI allele of each gen, we made use of a whole-body Dox-inducible system rosa26-rtTA [21].

### Treatments

Gene inactivation the OH-4-TAM treatment was induced systemically (intraperitoneal, IP, injection or supplemented in food) or locally (topical administration). For oral administration, *AurKB*lox/lox;- or *AurKB*lox/lox;Cre mice were fed with TAM-supplemented food (Harlan Laboratories Models). Intraperitoneal injection, over *Cdk1*+/lox;−, *Cdk1*lox/lox;Cre, *Plk1*+/+;Cre, *Plk1*lox/lox;Cre, *CDC20*+/lox;−, *CDC20*lox/lox;− or *CDC20*lox/lox;Cre mice, was implemented with TAM citrate salt (0.1 mg/g of animal body weight). For local topical treatment, *Cdk1*lox/lox;−, *Cdk1*lox/lox;Cre, *Plk1*+/+;Cre, *Plk1*+/lox;Cre, *Plk1*lox/lox;Cre, *CDC20*+/+;Cre, *CDC20*+/lox;Cre or *CDC20*lox/lox;Cre mice were painted on the ears with 50 µl TAM (5 mg/ml in 70% ethanol; H6278, Sigma-Aldrich) by using a micropipette.

Hyperproliferation in the skin tails, *Cdk1*lox/lox;−, *Cdk1*lox/lox;Cre, *Plk1*+/+;Cre, *Plk1*+/lox;Cre, *Plk1*lox/lox;Cre, *CDC20*+/+;Cre, *CDC20*+/lox;Cre or *CDC20*lox/lox;Cre mice were topically painted with 100 µl TAM (5 mg/ml in 70% ethanol) and 100 µl TPA (100 µg/ml in 100% acetone; P1585, Sigma-Aldrich) by using a micropipette.

To induce gene overexpression, *Plk1*+/+;rTA, *Plk1*+/KI;rTA, *AurKA*+/+;rTA or *AurKA*+/KI;rTA mice were fed with Dox (2 mg/ml supplemented with sucrose at 10 mg/ml) in the food for 10 days (*Plk1*+/+;rTA or *Plk1*+/KI;rTA mice) or 26 days (*AurKA*+/+;rTA or *AurKA*+/KI;rTA).

The following protocols were performed for intraperitoneal administration or the local topical induction of KO (a, b) and hyperproliferation (c, d).



**a**, **b** Intraperitoneal (IP) injection was performed the first 2 days of the experiment. Mice were euthanised after 4 (**a**, **b**), 6 (**b**) or 8 (**a**) days after treatment (red dotted lines) and epidermis from the tail and back was analysed. Topical treatment with TAM was performed on the outer surface of the ears every 2 days. Mice were euthanised after 7 days after treatment and the epidermis from the ears was analysed. **c**, **d** Topical treatment with TAM and TPA was performed on the surface of the tail every 2 days. Mice were sacrificed after 7 (**c**, **d**) or 10 (**d**) days after treatment and the epidermis from the tail was analysed.

The oral mucosa was also affected by topical administration due to the mice licking.

### Mice keratinocyte isolation and cell culture

Isolation and culture of mouse keratinocytes were slightly modified from Guinea-Viniegra et al. [63]. Briefly, tail or ears were obtained from euthanised mice. The bone was removed from the tail. The skin was rinsed once with 70% ethanol and then washed with PBS. The skin was then incubated in 0.75% trypsin for 1 h at 37 °C with 5% CO_2_. Trypsin was removed and the epidermis was separated with forceps. The epidermis was cut in pieces and trypsin was blocked with Rheinwald FAD medium. Epidermal cells were obtained by mechanical dissociation and filtered through a 100 μm cell strainer. Cells were either fixed in 70% ethanol or 3.7% paraformaldehyde for FC analyses (see below) or resuspended in culture media (17005034, Gibco) and plated on tissue culture plates previously coated for 1 h at 37 °C with coating matrix kit (R-011-K, Cascade Biologicals).

Gene deletion was induced in keratinocytes in vitro upon 48–96 h 100 nM OH-4-TAM (H7904, Sigma-Aldrich) in DMSO or 48 h with 100 nM of the Polo-like kinase 1 inhibitor (BI2536, Axon MedChem BV) in DMSO. Parallel control cultures were always subjected to the DMSO vehicle only.

### Human keratinocytes and cell culture

Ethical permission for this study was requested, approved and obtained from the Ethical Committee for Clinical Research of Cantabria Council, Spain (2014.166 and 2017.259). In all cases, human tissue material discarded after surgery was obtained with written consent presented by clinicians to the patients, and it was treated anonymously.

Human primary keratinocytes were cultured in the presence of a mouse fibroblast feeder layer (inactivated by mitomycin C), in Rheinwald FAD medium as described previously (10% foetal calf serum and 1.2 mM Ca^+2^) [64]. Low-culture passages (1–4) were utilised.

Primary keratinocytes were treated for 48 h with: 30 µM Blebbistatin (Blebb.; B-0560, Sigma- Aldrich) in DMSO or 0.5 µM Cytochalasin D (Cyto.; C-8273, Sigma-Aldrich) in DMSO. Parallel control cultures were always subjected to the DMSO vehicle.

### Clonogenicity assays

For clonogenicity assays, 3000–15,000 keratinocytes, both human or mouse, were grown in high-calcium FAD medium and plated per T6 well in triplicates. About 12–14 days later, the cultures were stained with rhodanile blue as described previously [36].

### Antibodies

The following antibodies were used: anti-Cdk1 (610038, BD Biosciences; immunohistochemistry (IHC)), anti-filaggrin (PRB-417P, Covance; immunofluorescence (IF)), anti-involucrin (RINVOL, 924401 Biolegend; FC and IF; SY3 [65], FC), anti-IgG (I-5381, Sigma-Aldrich; FC), anti-K1 (PRB-149P, Covance; FC), anti-K5 (SAB45016501, Sigma-Aldrich; IF), anti-K10 (C7284, Sigma-Aldrich; FC, sc-23877, Santa Cruz Biotechnology; IF and PRB-159P, Covance; IF), anti-K13 (sc-390982, Santa Cruz Biotechnology; IF), anti-K16 (LL025, Santa Cruz Biotechnolog; FC), anti-Ki67 (sc-7844, Santa Cruz Biotechnology; IF), anti-Plk1 (laboratory made; [18, 23] IHC), anti-phospho-histone H3 Ser10 (9701, Cell Signalling; IHC) and anti-PCNA (sc-56, Santa Cruz Biotechnology; IF).

The following secondary antibodies were used: Alexa Fluor® 488-conjugated goat anti-rabbit or anti-mouse IgG antibodies (Jackson; FC and IF), Alexa Fluor® 594-conjugated goat anti-rabbit or anti-mouse IgG antibodies (Jackson; IF).

### Live-cell Annexin V binding assay

Apoptosis was measured by CF647-Annexin V (FCCH100108, Millipore Bedford, MA, USA) binding assay in live cells following manufacturer instructions. As apoptotic positive control, mouse keratinocytes were irradiated with a 150 mJ/cm^2^ dose of UVB (312 nm; UPV CL-1000 Series UV crosslinker) as previously described [7].

### Flow cytometry

Keratinocytes were harvested, fixed and labelled for DNA content (PI), keratin K1, keratin K10, keratin K16 and involucrin as previously described [5]. All antibody stainings were controlled by the use of a similar concentration of isotype negative immunoglobulins (mouse IgGs or rabbit serum).

After staining cells were firmly resuspended and filtered through a 70–100 µM mesh to minimise the presence of aggregates and then analysed on a Becton Dickinson FACSCanto™ or a CytoFLEX (Bectam Coulter). Ten thousand events were gated and acquired.

The percent of positive cells was determined by use of a negative isotype Ig control and was represented in a bar histogram, KO cells relatively to control cells.

### Tissue histology and immunodetection

Dissected organs (skin from the back or tail and oral mucosa from tongue or cheek) were fixed in 10% buffered formalin (Sigma-Aldrich) and embedded in paraffin wax. Sections of 3 or 5 µm were stained with H/E.

IHC was performed with specific antibodies as previously described [21].

IF was slightly modified from Segrelles et al. [66]. Slides were deparaffinised with xylene and rehydrated with graded ethanol. Unmasking technique was performed by 10 min microwaving of slides in 0.01 M citrate buffer. Sections were incubated 30 min with NH_4_Cl to reduce autofluorescence. To block the Fc receptor, tissue sections were exposed to 8% goat serum for 60 min. Both primary and secondary antibodies were prepared in PBS/2% bovine serum albumin.

Scoring of positive cells on IHC or IF sections was performed by counting antibody positive cells by total nuclei in the field, based on DAPI staining. At least five fields were quantified per mice.

The size of basal nuclear area was determined by calculating the area of DAPI fluorescence per nucleus. Image J software was used to determine the area (in pixel^2^) of every nucleus in the basal layer. A minimum of 300 nuclei were analysed per mouse.

In situ terminal deoxynucleotidyl transferase-mediated deoxyuridine triphosphate-biotin nick-end labelling (TUNEL) was performed on tissue paraffin-embedded microsections according to the manufacturer’s instructions (Roche Diagnostics, Indianapolis, IN).

For determination of protein expression by western blotting, cells were washed with PBS, lysed and subjected to SDS-PAGE electrophoresis and western blotting as previously described [8].

### Confocal microscopy

For Supplementary Fig. 9, frozen tissue from human normal foreskin was microsectioned and 10-µm-thick frozen sections were collected on glass-slides Superfrost Plus (Thermo Scientific). They were fixed and permeabilised in methanol (−20 °C), and incubated with primary and secondary antibodies as previously described [36]. Images were obtained by confocal microscopy (Nikon A1R, Melville, NY, USA; 60 × numerical aperture 1.40) every 0.5 µm. Maximum intensity projection was performed for 2D and processed by NIS Elements software (AR, 3.2 64 bits; Nikon).

### Time-lapse video

Keratinocytes from *CDC20*lox/lox mice after a 48 h treatment with DMSO or TAM were treated with NucBlue Live ReadyProbes Reagent (Life Technologies) and filmed by time-lapse imaging for 24 h, photographed every 7 min. Phase-contrast and blue-fluorescence images were obtained by an epifluorescence microscope (NIKON Ti; 103NA 0.30) by an ORCA R2 camera.

### Statistical analyses

H/E staining, IHC or IF was randomly selected for the analyses. Data are presented as mean ± SD from at least three mice in freshly isolated keratinocytes (*N* = 3), three culture independent dishes in the in vitro conditions (*n* = 3) and two or three independent experiments as shown in each figure legend. Statistical methods appropriate for every figure, relative to the normal syngeneic mouse groups, for estimated variations and similarity among groups. Data sets were compared using an unpaired two-tailed Student’s *t* test when two data sets or one-way ANOVA when more than two data sets were analysed (GraphPad Prism 5). For multiple comparison, tests used were as follows: Tukey test, Newman–Keuls test or Boferroni test, depending on the dispersion of the data. A *P* value of <0.05 was considered statistically significant. In every case sample size was chosen accordingly. Damaged samples were excluded from analyses. Control and sample mice were chosen when possible to be littermates or at least of the same age. All animal samples analysed within the same group displayed similar results. Mice within each group were chosen by homogenous genotype, blind to the results. Microscope analyses on slide upon double blind were performed.

## Supplementary information

Suppl Fig 1

Suppl Fig 2

Suppl Fig 3

Suppl Fig 4

Suppl Fig 5

Suppl Fig 6

Suppl Fig 7

Suppl Fig 8

Suppl Fig 9

Suppl Fig 10
